# Scenario Projections of COVID-19 Burden in the US, 2024-2025

**DOI:** 10.1001/jamanetworkopen.2025.32469

**Published:** 2025-09-18

**Authors:** Sara L. Loo, Sung-mok Jung, Lucie Contamin, Emily Howerton, Samantha J. Bents, Harry Hochheiser, Michael C. Runge, Claire P. Smith, Erica C. Carcelén, Katie Yan, Joseph C. Lemaitre, Emily Przykucki, Clifton D. McKee, Koji Sato, Allison L. Hill, Matteo Chinazzi, Jessica T. Davis, Clara Bay, Alessandro Vespignani, Shi Chen, Rajib Paul, Daniel Janies, Jean-Claude Thill, Sean M. Moore, T. Alex Perkins, Ajitesh Srivastava, Majd Al Aawar, Kaiming Bi, Shraddha Ramdas Bandekar, Anass Bouchnita, Spencer J. Fox, Lauren Ancel Meyers, Przemyslaw Porebski, Srinivasan Venkatramanan, Bryan Lewis, Jiangzhuo Chen, Madhav Marathe, Michal Ben-Nun, James Turtle, Pete Riley, Katriona Shea, Cécile Viboud, Justin Lessler, Shaun Truelove

**Affiliations:** 1 Johns Hopkins University, Baltimore, Maryland; 2 University of North Carolina at Chapel Hill, Chapel Hill; 3 University of Pittsburgh, Pittsburgh, Pennsylvania; 4 Princeton University, Princeton, New Jersey; 5 National Institutes of Health Fogarty International Center, Bethesda, Maryland; 6 US Geological Survey, Laurel, Maryland; 7 Penn State University, University Park, Pennsylvania; 8 Northeastern University, Boston, Massachusetts; 9 University of North Carolina at Charlotte, Charlotte; 10 University of Notre Dame, Notre Dame, Indiana; 11 University of Southern California, Los Angeles; 12 School of Public Health, The University of Texas Health Science Center at Houston, Houston; 13 University of Texas at Austin, Austin,; 14 University of Texas at El Paso, El Paso; 15 University of Georgia, Athens, Georgia; 16 University of Virginia, Charlottesville; 17 Predictive Science Inc, San Diego, California

## Abstract

**Importance:**

COVID-19 remains a disease with high burden in the US, prompting continued debate about optimal targets for annual vaccination.

**Objective:**

To project COVID-19 burden in the US for April 2024 to April 2025 under 6 scenarios of immune escape (20% and 50% per year) and levels of vaccine recommendation (no recommendation, vaccination for individuals at high risk only, vaccination for all eligible groups) and to assess the potential benefit of vaccine recommendations in reducing disease burden.

**Design, Setting, and Participants:**

For this decision analytical model, the US Scenario Modeling Hub, a collaborative modeling effort, convened 9 teams to provide scenario projections of US COVID-19 hospitalizations and deaths for April 2024 to April 2025, under 6 scenarios combining levels of immune escape and possible vaccine recommendations.

**Exposure:**

Annually reformulated vaccines were assumed to be 75% effective against hospitalization for variants circulating on June 15, 2024, and available on September 1, 2024. Age- and state-specific coverage was assumed to be as reported in September 2023 to April 2024.

**Main Outcomes and Measures:**

Ensemble estimates were made for weekly COVID-19 hospitalizations and deaths. Projections are presented for relative and absolute prevented hospitalizations and deaths averted due to vaccination over the April 2024 to April 2025 period.

**Results:**

For the US population (332 million, with an estimated 58 million aged ≥65 years), COVID-19 was expected to cause 814 000 (95% projection interval [PI], 400 000-1.2 million) hospitalizations and 54 000 (95% PI, 17 000-98 000) deaths for April 2024 to April 2025, comparable in magnitude to the prior year. Vaccination of high-risk groups only was projected to reduce hospitalizations (compared to no vaccination recommendation) by 76 000 (95% CI, 34 000-118 000) and deaths by 7000 (95% CI, 3000-11 000) across both immune escape scenarios. Compared with vaccinating high-risk groups only, a universal vaccine recommendation was projected to provide direct and indirect benefits, further preventing 11 000 hospitalizations and 1000 deaths in those aged 65 years and older.

**Conclusions and Relevance:**

In this decision analytical modeling study of COVID-19 burden in the US in 2024 to 2025, ensemble projections suggested that although vaccinating high-risk groups had substantial benefits in reducing disease burden, maintaining the vaccine recommendation for all individuals had the potential to save thousands more lives. Despite divergence of projections from observed disease trends in 2024 to 2025—possibly driven by variant emergence patterns and immune escape—averted COVID-19 burden due to vaccination was robust across immune escape scenarios, emphasizing the substantial benefit of broader vaccine availability for all individuals.

## Key Points

**Question:**

To what extent could COVID-19 vaccination reduce the likely disease burden in the US from April 2024 to April 2025?

**Findings:**

In this decision analytical model using ensemble-based projections of COVID-19 hospitalizations and related deaths under plausible assumptions of immune escape and vaccine recommendations, the projected disease burden during April 2024 to April 2025 was similar in magnitude to the prior year. Vaccination of all individuals was projected to reduce 10% to 20% of COVID-19 burden compared with no vaccination recommendation, with additional indirect benefits to individuals aged 65 years and older compared with vaccinating high-risk groups only.

**Meaning:**

These findings suggest that vaccines can be an effective tool for limiting COVID-19 burden, with a universal vaccine recommendation potentially saving thousands more lives through direct and indirect effects.

## Teaser

This decision analytical model presents projected COVID-19 hospitalizations and deaths under 6 different vaccination recommendation and immune escape scenarios in the US from April 2024 to April 2025.

## Introduction

COVID-19 continues to be a disease of substantial public health concern, with a higher risk of death among hospitalized patients than seasonal influenza during fall/winter 2023 to 2024.^1^ The Advisory Committee on Immunization Practices develops recommendations on COVID-19 vaccine use in the US (including dosage and target populations), guiding the Centers for Disease Control and Prevention (CDC) in setting immunization schedules.^2^ However, the population impact of these vaccination campaigns, and the benefits of focusing on those outside of high-risk groups, remain uncertain. Scenario modeling can compare alternative futures, enabling projections of population-level effects and providing evidence to inform vaccine recommendation decisions.

Since December 2020, the US Scenario Modeling Hub^3^ has generated 18 rounds of ensemble-based projections at state and national levels to inform COVID-19 policy.^4^ These projections were generated by aggregating outcomes from multiple models designed to evaluate the same sets of scenarios; this approach has been shown to enhance the reliability of predictions compared with the performance of individual models.^5^ Ahead of Advisory Committee on Immunization Practices discussions on updated COVID-19 booster vaccinations in June 2024, the US Scenario Modeling Hub developed a new round of projections to compare the potential advantages of possible annual vaccine recommendations during 2024 to 2025, aiming to inform decision-making.

Here, we present yearlong projections of COVID-19 hospitalizations and deaths in the US released in spring 2024 under various viral evolution and vaccination scenarios. By comparing outcomes across scenarios, we assess the direct and indirect benefits of different vaccine recommendations in 2024 to 2025. Additionally, the 2024 to 2025 respiratory virus season has concluded, so we also compare our projections with the observed trajectory of COVID-19 over this time period and discuss the potential drivers behind any discrepancies.

## Methods

This decision-analytical model was deemed exempt from review and informed consent because it used routinely collected surveillance data that were processed anonymously at all stages, per 45 CFR 46. To project the potential benefits of possible recommendations of vaccination strategy in the US (at national and state levels) for 2024 to 2025, the US Scenario Modeling Hub convened 9 teams to provide projections of COVID-19 hospitalizations and deaths for April 28, 2024, to April 26, 2025. Teams provided projections under 6 scenarios across 2 axes of uncertainty: immune escape and annual vaccine recommendations (following scenario design best practices^6^). Teams calibrated their models using weekly COVID-19 hospitalizations and deaths from the US National Healthcare Safety Network and the National Center for Health Statistics, respectively,^7,8^ with calibration specifics (eg, timeframe for calibration or inclusion of additional data sources^9,10,11^) left to the teams’ discretion.

Scenarios representing 3 possible recommendations for the use of annually reformulated vaccines were considered: (1) no vaccine recommendation (only vaccination of vaccine-naive children aging into eligibility at age 6 months), (2) recommendation for only high-risk groups (adults aged ≥65 years and those with underlying conditions^12^), and (3) recommendation for all eligible groups (everyone aged ≥6 months). In all scenarios, all models assumed vaccines were reformulated to match the predominant variants circulating on June 15, 2024, and became available on September 1, 2024, with vaccine effectiveness of 75% against hospitalization at the population level at the time of availability^13^ (eFigure 1 in Supplement 1). State-specific annual vaccine uptake in respective eligible groups was assumed to be the same as reported in September 2023 to April 2024,^14^ incorporating state- and age-specific population size and vaccine coverage for high- and low-risk groups provided by the CDC.^14^ Individual model assumptions were left to each teams’ scientific discretion (eTable 1 in Supplement 1), including variation in vaccine effectiveness against other outcomes or across specific groups (with guidelines on the relative risk of hospitalization for high-risk groups) and intrinsic immune waning. Although teams were directed to incorporate infection-acquired immunity, the specific implementation details (including protection effectiveness against each outcome and the duration of immunity) were left to the discretion of each team without imposing any constraints on assumptions (eTable 1 in Supplement 1).

Scenarios considered immune escape due to viral evolution that reduces protection against infection (from vaccination or prior infection) at a specified annual rate of 20% (low) or 50% (high) per year. For instance, under the 20% rate, a vaccine formulated based on a strain circulating in April 2024 would be 20% less effective against symptomatic infection with strains circulating in April 2025. Models assumed either that SARS-CoV-2 evolved away from existing immunity at a constant rate or through frequent stepwise changes (≥4 variants per year). Immune escape against severe outcomes was at the teams’ discretion. All scenario details are summarized in the Table. Modeling teams were instructed to provide projections for hospitalizations and deaths in 50 states, the District of Columbia, and at the national level under these 6 scenarios.

**Table.** COVID-19 Projection Scenario Definitions for April 28, 2024-April 26, 2025

	Low immune escape at constant rate of 20% per year	High immune escape at constant rate of 50% per year
No vaccine recommendation Only naive children aging into eligibility get vaccinated	Low immune escape, no vaccine recommendation	Low immune escape, no vaccine recommendation
Reformulated annual vaccination recommended for high-risk groups (≥65 y and those with underlying risk factors) Vaccine becomes available September 1 to high-risk groups Reformulated vaccine has 75% VE against hospitalization at time of delivery on September 1^a^ Uptake in high-risk groups (individuals aged ≥65 y or with underlying risk factors) is the same as seen for 2023-2024 reformulated vaccine Uptake in naive children aging into eligibility is same as seen for 2023-2024 Uptake in all other groups is negligible	Low immune escape, high-risk only	High immune escape, high-risk only
Reformulated annual vaccination recommended for all currently eligible groups Vaccine becomes available September 1 Reformulated vaccine has 75% VE against hospitalization at time of delivery on September 1^a^ Coverage saturates at levels of the 2023-2024 booster for all age/risk groups (approximately 21% of adults nationally, 39% of those aged ≥65 y, 32% of high-risk individuals)	Low immune escape, All	High immune escape, All

Abbreviation: VE, vaccine effectiveness.

^a^ VE was defined as the vaccine effectiveness against COVID-19 hospitalization at the time of vaccine release on September 1, 2024. This assumption was based on a study from Denmark^13^ and was equivalent to a vaccine trial performed on September 1, 2024, in populations with varying levels of prior immunity at the point of enrollment. This equates to vaccinated individuals having a 75% reduced risk of hospitalization compared with unvaccinated individuals on average, if VE was estimated a few days after release.

### Statistical Analysis

Individual model projections were combined to produce ensemble projections using a trimmed linear opinion pool method,^15^ and estimates of burden averted under different mitigating scenarios were obtained using meta-analysis approaches.^16^ All analyses were conducted using R software version 4.4.1 (R Project for Statistical Computing).

## Results

### General Magnitude of Burden

**Figure 1.**
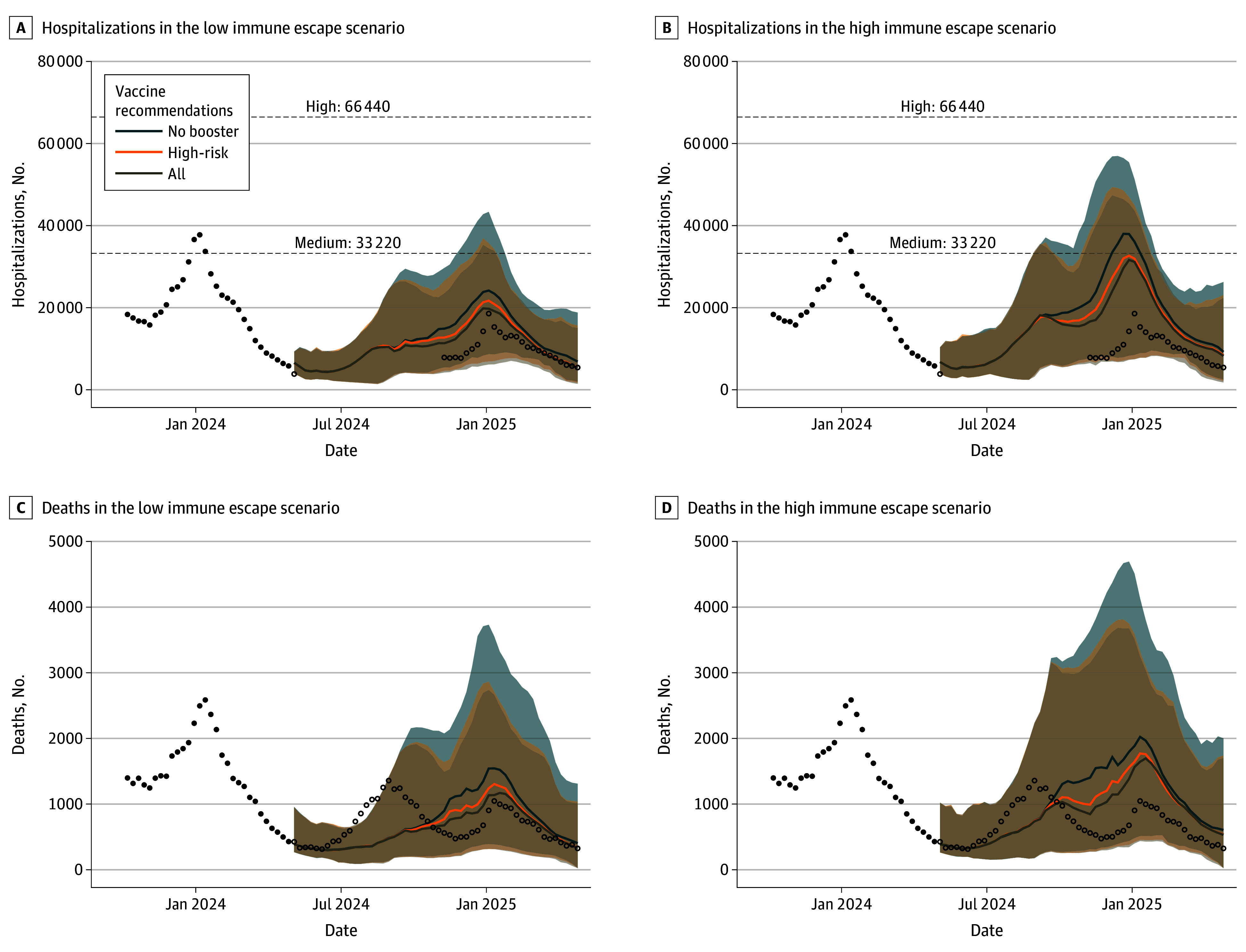
Weekly Projections of COVID-19 Hospitalizations and Deaths in the United States During April 28, 2024, to April 26, 2025, Across 6 Scenarios of Immune Escape and Vaccination Recommendations Ensemble projections are based on 9 models, and historical data are shown as black points. Unfilled data points are observed data after the projection period; National Healthcare Safety Network hospitalization data are shown only where the fraction of reporting was greater than 75%, showing the gap in reliable data during this time (May 5, 2024, to November 2, 2024). Projection curves are colored based on vaccine recommendations, with curves representing the median of the ensemble projection and shading showing the 90% projection interval. Dashed lines indicate the Centers for Disease Control and Prevention thresholds for high and medium hospital admission levels (>20 and 10-20 weekly hospitalizations per 100 000, respectively).

All projections were released on a public website in June 2024, ahead of vaccine recommendations discussion for that year.^3^ Based on an ensemble of projections from 9 contributing models under 6 scenarios incorporating different assumptions about immune escape levels and vaccine recommendations, we projected that COVID-19 burden in the US for April 28, 2024, to April 26, 2025, would remain comparable in magnitude to that of the previous year. In all 6 scenarios, ensemble projections suggested that national COVID-19 hospitalizations would remain below the CDC threshold for low hospital admission levels (defined as <10 weekly hospitalizations per 100 000^17^) throughout spring of 2024, followed by an increase in late summer and fall toward a peak in December 2024 to mid-January 2025 comparable in magnitude to, or lower than, the previous winter’s 2023 to 2024 peak. In the high immune escape scenarios, a projected summer peak was more pronounced. In general, throughout the projection period, weekly national hospitalization projections remained below the high CDC hospital admission levels (defined as >20 weekly hospitalizations per 100 000^17^) across all scenarios (Figure 1).

In the worst-case scenario (the scenario expected to generate the highest disease burden, namely, high immune escape and no vaccine recommendations), ensemble projections predicted 931 000 (95% projection interval [PI], 0.5 million to 1.3 million) cumulative hospitalizations by April 26, 2025 and 62 000 (95% PI, 18 000 to 115 000) deaths. In the best-case scenario (the scenario expected to generate the lowest disease burden, namely, low immune escape and vaccine recommendations for all individuals), the ensemble projected 550 000 (95% PI, 296 000 to 832 000) hospitalizations and 42 000 (95% PI, 13 000 to 72 000) deaths. In the scenario specifications most similar to the April 2023 to April 2024 time period (high immune escape and a universal vaccine recommendation), the ensemble projected 814 000 (95% PI, 400 000 to 1.2 million) hospitalizations and 54 000 (95% PI, 17 000 to 98 000) deaths. Most severe COVID-19 outcomes were projected to occur among individuals aged 65 years and older (51%-62% of total hospitalizations and 84%-87% of total deaths across all scenarios).

### Vaccine Impact

**Figure 2.**
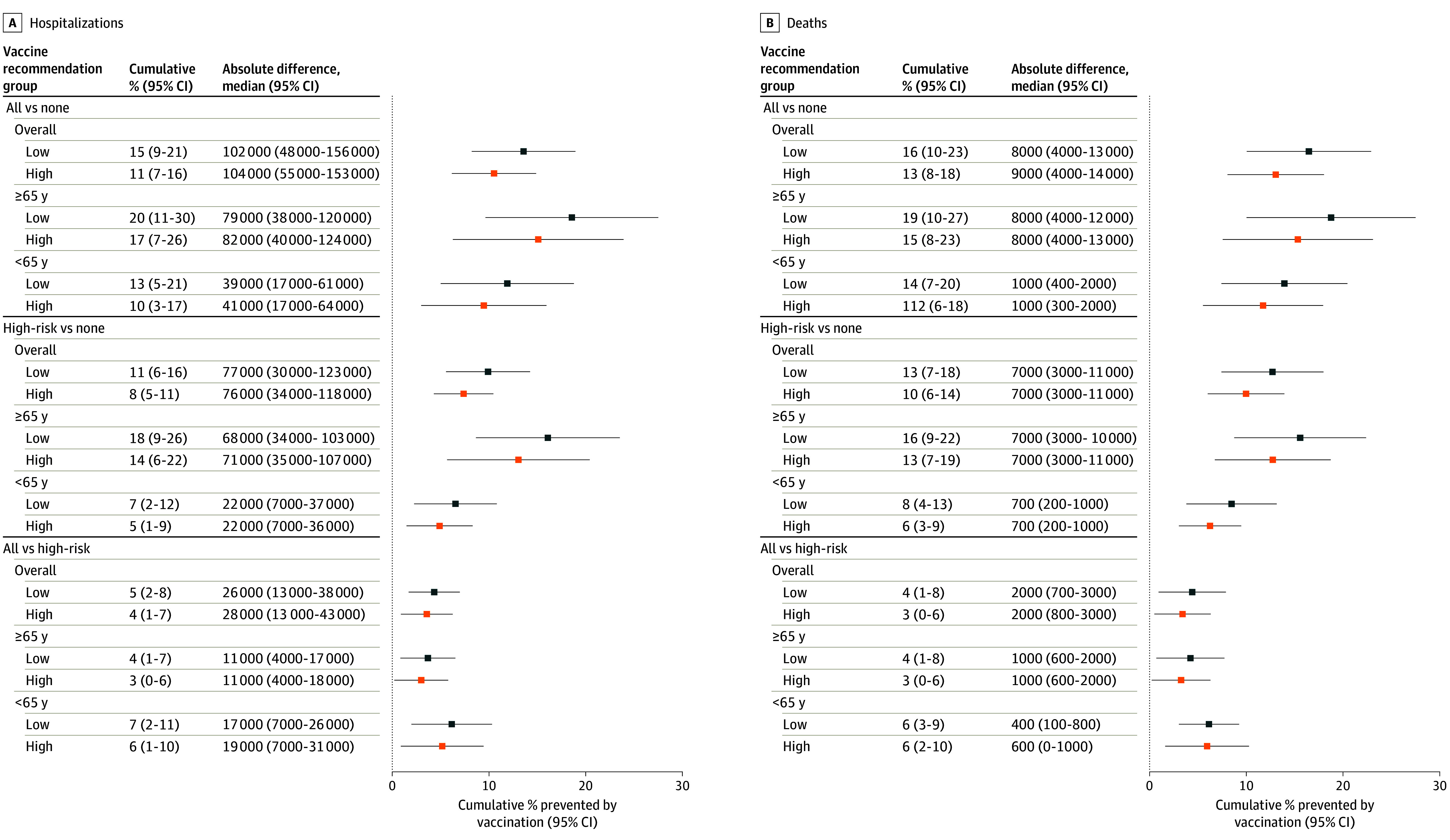
Cumulative Percentage and Absolute Prevented Hospitalizations and Deaths Averted Under Different Vaccination Recommendations and Rates of Immune Escape for April 2024 to April 2025 Low immune escape was defined as 20% per year and high, 50% per year.

Vaccine recommendations for high-risk groups and all individuals were projected to substantially reduce the disease burden compared with no vaccine recommendation across immune escape scenarios. Under low and high immune escape scenarios respectively, vaccination of high-risk groups was projected to reduce hospitalizations by 11% (95% CI, 6%-16%) and 8% (95% CI, 5%-11%) and deaths by 13% (95% CI, 7%-18%) and 10% (95% CI, 6%-14%); targeting all ages was projected to reduce hospitalizations by 15% (95% CI, 9%-21%) and 11% (95% CI, 7%-16%) and deaths by 16% (95% CI, 10%-23%) and 13% (95% CI, 8%-18%). In absolute numbers, vaccinating high-risk groups was projected to result in 76 000 (95% CI, 34 000-118 000) fewer hospitalizations and 7000 (95% CI, 3000-11 000) fewer deaths nationally in the high immune escape scenario. Expanding vaccination to all ages would further reduce burden, with 104 000 (95% CI, 55 000-153 000) hospitalizations and 9000 (95% CI, 4000-14 000) deaths averted under high immune escape scenarios (similar reductions were observed in low immune escape scenarios) (Figure 2).

Expanding recommendations to all ages was projected to prevent an additional 28 000 (95% CI, 13 000-43 000) hospitalizations and 2000 (95% CI, 800-3000) deaths compared with vaccinating high-risk groups only in the high immune escape scenario. Although most of the overall projected vaccine benefits (both direct and indirect) resulted from a reduction in burden among individuals aged 65 years and older, universal vaccine recommendations were projected to produce moderate indirect benefits to all age groups. Indirect effects in adults aged 65 years and older were calculated by comparing the burden under universal and high-risk-only recommendations (both of which include this age group), across projections that were paired on all other parameters. In these comparisons, the universal vaccine recommendation was projected to further reduce burden by 3% to 4% in the group of adults aged 65 years and older (approximately 11 000 hospitalizations and 1000 deaths averted).

### Comparisons With Observed 2024 to 2025 COVID-19 Burden in the US

**Figure 3.**
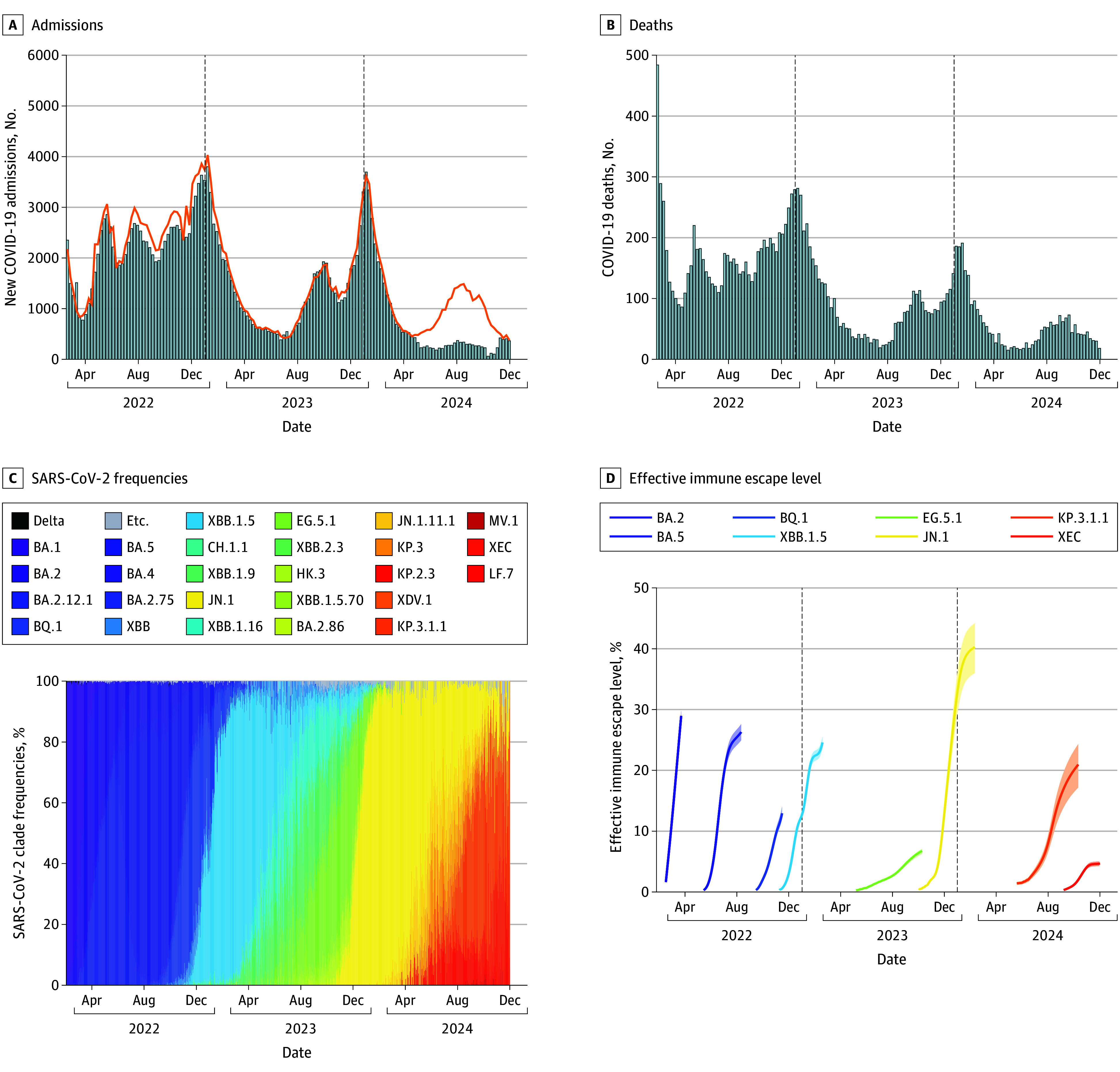
Weekly COVID-19 Hospitalizations, Deaths, and SARS-CoV-2 Clade Characteristics in New York State, April 2022 to December 2024 A, Bars indicate reported hospitalizations from the Centers for Disease Control and Prevention (CDC) National Healthcare Safety Network data^7^; line, hospitalization data from New York state COVID-19 hospitalization dashboard using the Health Electronic Response Data System^9^, showing a substantial reduction in reporting to CDC during the summer of 2024. B, Weekly deaths from the National Center for Health Statistics.^8^ C, Weekly frequencies of SARS-CoV-2 clades obtained from Nextstrain.^10^ D, Effective immune escape level of dominant clades (defined as clades that reached a frequency >50% of sequenced samples). Effective immune escape level was calculated by multiplying the estimated percentage relative immune escape level with the reported proportion of each clade (C). Relative immune escape level was estimated based on the relative growth advantage per week of the clade compared to the preexisting dominant clade, assuming the same transmissibility between the 2 variants and a generation interval of 3.5 days^11^. California data are presented in eFigure 2 in Supplement 1.

Although our projections were initially released in June 2024, the end of the 2024 to 2025 respiratory virus season provided an opportunity to compare projections with the observed COVID-19 trajectories and assess the validity of our ensemble estimates. Due to a pause in National Healthcare Safety Network reporting between May 4 and November 1, 2024, during which less than 75% of hospitals submitted new admission data, comprehensive comparisons for hospitalizations were limited. Nevertheless, reported deaths (Figure 1) and alternative robust data streams (including state health department data, eg, New York^9^ and California^18^) (Figure 3A; eFigure 2 in Supplement 1) indicated that the US experienced a notable COVID-19 wave during the summer of 2024. This wave peaked in mid- to late August, followed by a marked decline in transmission through the fall, and a smaller wave of hospitalizations in January 2025. These trends diverged from our ensemble projections, which expected that COVID-19 would produce the most burden during December 2024 to January 2025, with some potential increases in late summer of 2024. This discrepancy coincided with a higher effective immune escape level (defined as the product of estimated percentage relative immune escape levels and the proportion of each clade) of the SARS-CoV-2 variant circulating in the summer of 2024, followed by a lower level in the winter, in contrast to patterns observed in 2023 2024 (Figure 3D; eFigure 2 in Supplement 1). Despite this divergence, the comparison between observation and projections shows robust accuracy under scenarios in which boosters were recommended for all individuals (reflecting the real-world policy implemented in 2024-2025^19^). We found that the coverage of the ensemble was close to normal values for weekly COVID-19 deaths throughout the projection period (eg, the 95% PIs captured close to 95% of observations) (eFigure 3 in Supplement 1), supporting the robustness of our ensemble projections.

## Discussion

In this decision analytical model, our ensemble projections, released ahead of vaccine recommendations in 2024 to 2025, suggested that COVID-19 would remain a persistent public health burden in the US, with a peak in hospitalizations and death in the winter expected in late December to early January, comparable in magnitude to 2023to 2024 across all 6 scenarios. Under 2 strategies of vaccine recommendations, targeted at either high-risk individuals or all eligible individuals, we projected substantial burden averted from continuing to recommend updated vaccination for COVID-19 in fall 2024, across immune escape assumptions.

Although the potential impact of vaccinating high-risk groups on disease burden was substantial, extending recommendations to all eligible groups was projected to further reduce hospitalizations and deaths, including among individuals aged 65 years and older. This finding suggests substantial indirect benefits of universal vaccination and the continued value of broad vaccine recommendations. However, the extent of these indirect benefits depends on vaccine uptake among low-risk individuals and the assumed vaccine effectiveness against infection and transmission in each individual model. Our vaccine uptake assumptions were based on observed patterns in 2023 to 2024,^14^ so the projected indirect effects would be even more pronounced, given the observed modest increase in uptake in 2024 to 2025 across age and risk groups (eFigure 4 in Supplement 1).

We emphasize that the value in our scenario projections is not in their ability to forecast the future, but rather in their robust comparison of vaccination scenarios. However, we recognize that the observed timing of COVID-19 hospitalizations during 2024 to 2025 in the US diverged substantially from all of our scenario projections, although death projections across 2024 to 2025 had good coverage. This divergence could result from a number of factors. Observations suggest there was a notable COVID-19 epidemic wave in the summer of 2024, which was not accurately captured by the ensemble projections. Our scenarios assumed a constant rate of immune escape over time, based on the variant dynamics that were observed following the initial Omicron wave (winter 2021 to spring 2022). Although this assumption might hold for some years, it could substantially fall short in other years, as evolutionary dynamics follow irregular patterns. Specifically, we saw the emergence of multiple variants with high relative growth advantage and effective immune escape continually throughout 2022, coinciding with large peaks in hospitalizations and deaths in summer and winter (Figure 3; eFigure 5 in Supplement 1). However, 2023 was characterized by a relatively low immune escape variant emerging during the summer, flanked by high immune escape variants in winter 2022 and winter 2023 (XBB.1.5 in December 2022 and JN.1 in December 2023). This coincided with a more attenuated summer season compared with winter seasons. For 2024, this pattern appears to have diverged again, with a higher immune escape variant emerging during the summer (KP.3.1.1) but a very low immune escape variant during winter 2024 (XEC), coinciding with a notably higher summer peak than winter peak. In our projection, higher constant immune escape drives more distinct late summer peaks, as evidenced by our 2 high immune escape scenarios, whereas lower immune escape is more likely to maintain some durability of population immunity, limiting large summer increases and leading to a more gradual increase from late summer through winter. These dynamics of immune escape, in concert with the complex interacting dynamics of transmission and waning immunity, appear to be a strong factor in driving surges in transmission and the apparent seasonality of COVID-19.^20,21,22^

It is important to note that, given the nature of projections, predicting the likely course of the epidemic within a defined scenario rather than forecasting the future, any potential divergence between projected and observed outcomes does not undermine the robustness of the projections. Nevertheless, understanding the drivers of COVID-19 seasonality and viral evolution could explain the discrepancies observed during the late summer and early winter of 2024 and help refine future scenarios and modeling approaches.

### Limitations

Given the nature of forward-looking modeling efforts, our projections are likewise subject to some limitations. First, specifications of COVID-19 seasonality may affect our projected vaccine benefit estimates. The absolute number of hospitalizations and deaths averted by vaccination may depend on the timing of both vaccine administration and transmission waves. Immunity acquired during a notable summer wave in 2024—occurring prior to the start of the vaccination campaign (September 2024) and not captured in our projections—might lead to an overestimation of vaccine benefits. Nevertheless, the relative differences in disease burden reductions across scenarios with different vaccine recommendations are likely to remain robust.

Second, the variability in variant dynamics and immune escape and static assumptions of the scenarios may have impacted calibration of each model. With our scenario assumption of continuous immune escape, other seasonal forcings in individual models might be calibrated to compensate for this discrepancy in immune escape effects. This overestimated or underestimated seasonality could lead to exaggerated increases in transmission in the projection period.

Third, in the absence of the CDC vaccine recommendation, vaccines would not be covered by insurance, and uptake among children newly aging into eligibility (ie, age 6 months) may diverge from levels observed in the prior season—levels that served as the basis for vaccine coverage in the projection period.^23^ Nevertheless, young children make up a small share of the total population, and vaccine uptake in this group has remained low (approximately 6% in children aged 6 months to 4 years in 2023-2024,^23^ a prior season used for indexing coverage in our projections). Hence, any resulting discrepancies in these levels of vaccine coverage are expected to have minimal impact at the population level and are unlikely to affect our main results.

Additionally, the potential effect resulting from variations in the details of modeling approaches (eg, assumptions on infection-acquired immunity and seasonal variation in intrinsic transmissibility) was not quantified due to the multiteam nature of the Scenario Modeling Hub framework. Moreover, disentangling the effect of any single factor can be nontrivial, given the numerous interacting factors that shape COVID-19 trajectories.

## Conclusions

Despite unclear variant and seasonal dynamics, our ensemble scenario-based projections in this decision analytical model provide support for decisions about COVID-19 vaccine recommendations and suggest that vaccines would remain a critical tool to limit the burden of COVID-19 in the US during 2024 to 2025, particularly among high-risk individuals. Furthermore, while focusing on vaccination among individuals at the highest risk for severe outcomes, including those aged 65 and older and those with comorbidities, remains an effective ongoing strategy, our scenario projections demonstrate that maintaining the recommendation for all individuals to receive reformulated vaccines has the potential to save thousands more lives through both direct and indirect effects. We expect that despite divergence between our projections and observed data, the effect of extending recommendations to all would prolong protection across age groups, particularly in light of lower rates of immune escape during the 2024 winter. With the projected potential for COVID-19 burden similar to severe influenza season in the most optimistic scenario,^24^ modeling and surveillance efforts will continue to inform vaccine recommendations and aid in mitigating disease impact.

## Notes

Supplement 1. **eFigure 1.** Time-varying vaccine effectiveness against hospitalization under assumptions on the rates of immune waning and immune escape. **eFigure 2.** Weekly COVID-19 hospitalizations, deaths, and SARS-CoV-2 clade characteristics in California, April 2022-December 2024 **eFigure 3.** Quantile-quantile (QQ) plot for assessing the performance of ensemble projection regarding weekly COVID-19 deaths in the United States **eFigure 4.** Comparison between the assumed and observed uptake levels of COVID-19 vaccines at the national level in the United States **eFigure 5.** Observed hospitalizations and death target data from April 28, 2023 to April 26, 2025 in the United States **eTable.** Detailed description of individual models
eReferences.

Supplement 2.
Data Sharing Statement
