# Supplementary material for: Scenario Projections of COVID-19 Burden in the US, 2024-2025
Source: JAMA Netw Open. 2025 Sep 18;8(9):e2532469. doi: 10.1001/jamanetworkopen.2025.32469 (PMC12447233; doi:10.1001/jamanetworkopen.2025.32469)
Supplement: Supplement 1. — eFigure 1. Time-varying vaccine effectiveness against hospitalization under assumptions on the rates of immune waning and immune escape. eFigure 2. Weekly COVID-19 hospitalizations, deaths, and SARS-CoV-2 clade characteristics in California, April 2022-December 2024 eFigure 3. Quantile-quantile (QQ) plot for assessing the performance of ensemble projection regarding weekly COVID-19 deaths in the United States eFigure 4. Comparison between the assumed and observed uptake levels of COVID-19 vaccines at the national level in the United States eFigure 5. Observed hospitalizations and death target data from April 28, 2023 to April 26, 2025 in the United States eTable. Detailed description of individual models eReferences. [file jamanetwopen-e2532469-s001.pdf]

Supplemental Online Content

Loo SL, Jung S, Contamin L, et al. Scenario projections of COVID-19 burden in the US, 2024-2025. *JAMA Netw Open*. 2025;8(9):e2532469.
doi:10.1001/jamanetworkopen.2025.32469

eFigure 1. Time-varying vaccine effectiveness against hospitalization under assumptions on the rates of immune waning and immune escape.
eFigure 2. Weekly COVID-19 hospitalizations, deaths, and SARS-CoV-2 clade characteristics in California, April 2022-December 2024
eFigure 3. Quantile-quantile (QQ) plot for assessing the performance of ensemble projection regarding weekly COVID-19 deaths in the United States
eFigure 4. Comparison between the assumed and observed uptake levels of COVID-19 vaccines at the national level in the United States
eFigure 5. Observed hospitalizations and death target data from April 28, 2023 to April 26, 2025 in the United States
eTable. Detailed description of individual models
eReferences.

This supplemental material has been provided by the authors to give readers additional information about their work.

© 2025 Loo SL et al. *JAMA Network Open*.

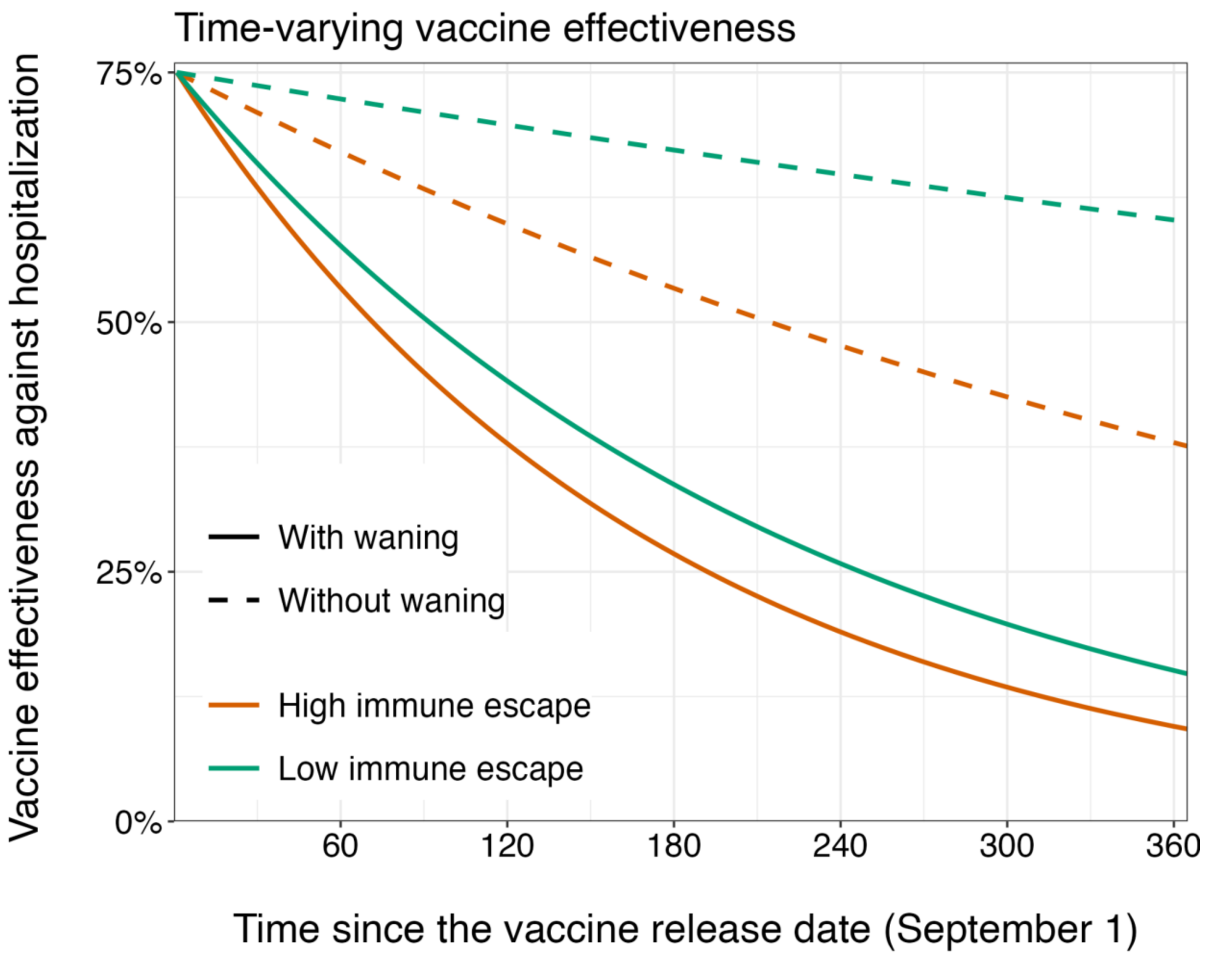

eFigure 1. Time-varying vaccine effectiveness against hospitalization under assumptions on the rates of immune waning and immune escape. Vaccine effectiveness against hospitalization which is modeled to decline over time following (i) immune escape and (ii) waning immunity. Immune escape as a result of antigenic drift of circulating SARS-CoV-2 variants was assumed to occur at constant rates corresponding to 20% and 50% reductions in protection per year for low (green) and high (orange) immune escape scenarios, respectively. Waning immunity was assumed to follow an exponential decay pattern with a 6-month half-life, reflecting the assumptions of most individual models (solid lines). The dashed lines represent scenarios that do not account for waning immunity. The time-varying vaccine

© 2025 Loo SL et al. *JAMA Network Open*.

effectiveness against hospitalization in each model may differ based on their specific assumptions regarding the decay pattern and half-time of immunity.

© 2025 Loo SL et al. *JAMA Network Open*.

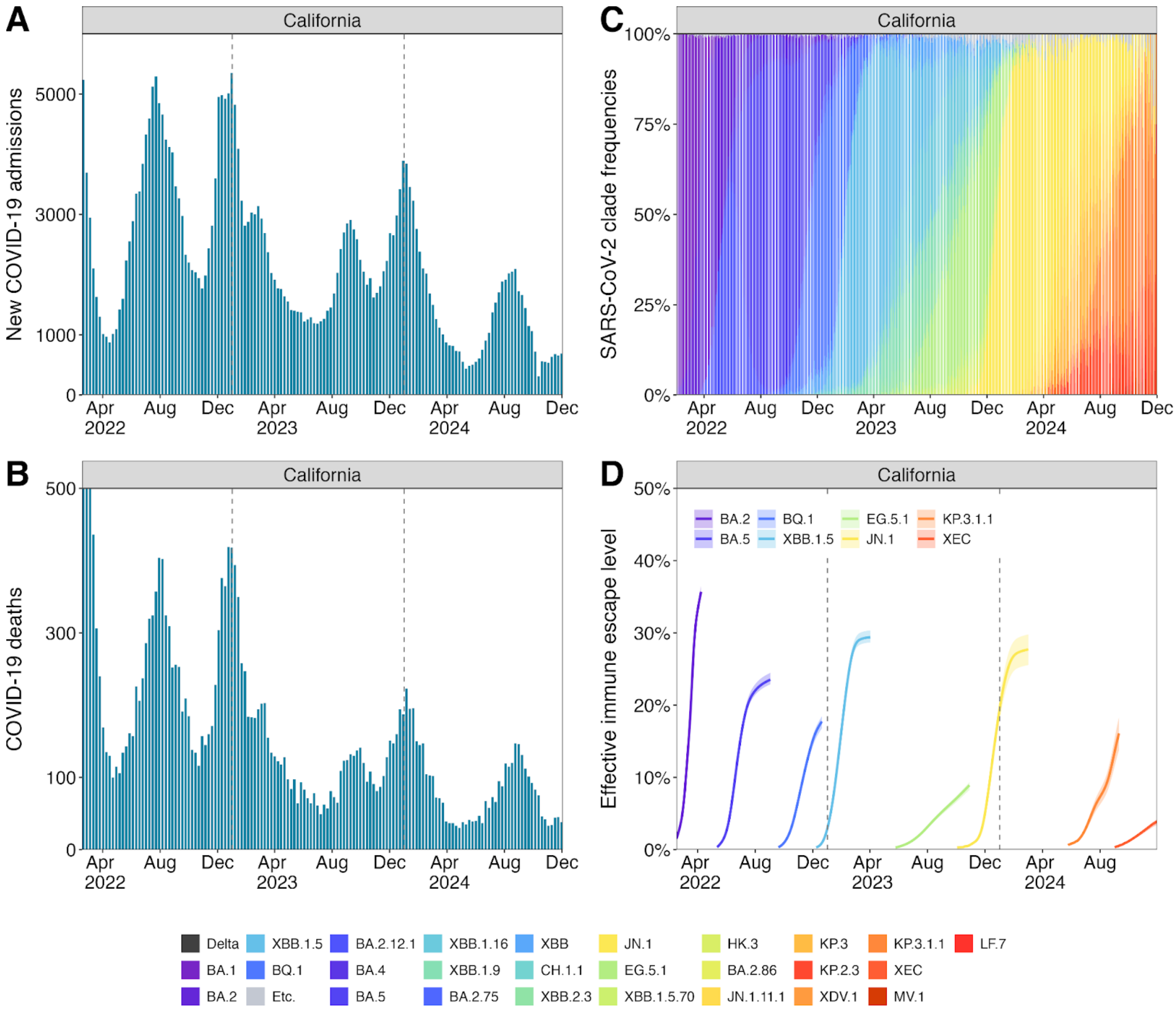

eFigure 2. Weekly COVID-19 hospitalizations, deaths, and SARS-CoV-2 clade characteristics in California, April 2022–December 2024. (A) Weekly new COVID-19 admissions in California. Blue bars indicate reported hospitalizations from the CDC National Healthcare Safety Network data[1]. (B) Weekly deaths from the National Center for Health Statistics[2]. (C) Weekly frequencies of SARS-CoV-2 clades obtained from Nextstrain[3]. (D) Effective immune escape level of dominant clades (defined as clades that reached a frequency greater than 50% of sequenced samples). Effective immune escape level was calculated by multiplying the estimated percent relative immune escape level with the reported proportion of each clade (panel C). Relative immune escape level was estimated based on the relative growth advantage per week of the clade compared to the pre-existing dominant clade, assuming the same transmissibility between the two variants and a generation interval of 3.5 days[4].

© 2025 Loo SL et al. *JAMA Network Open*.

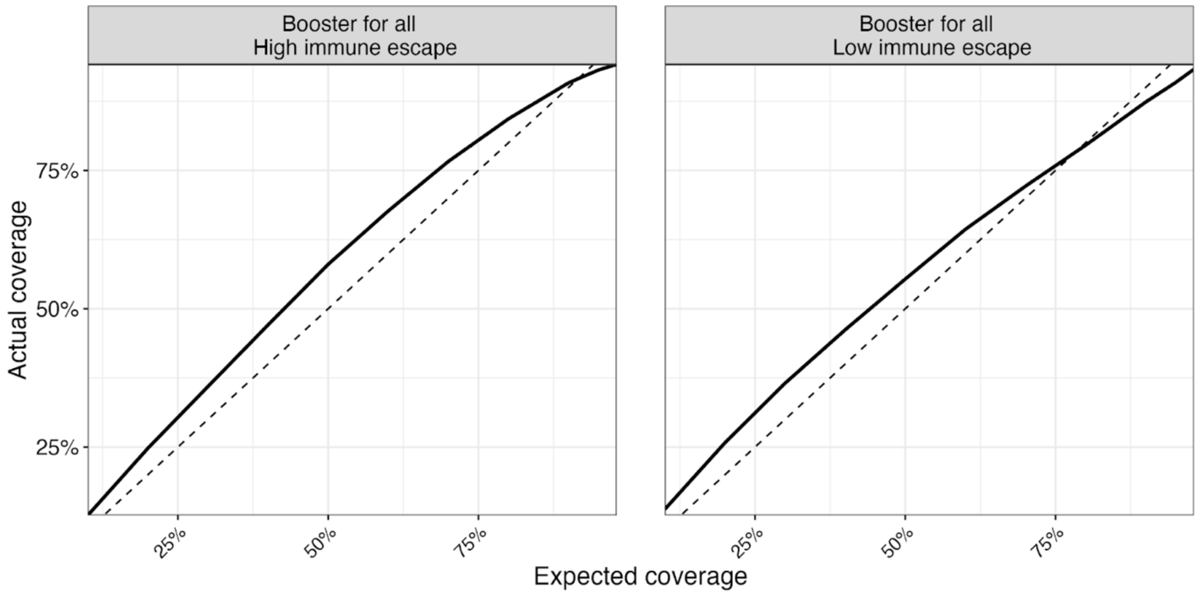

eFigure 3. Quantile-quantile (QQ) plot for assessing the performance of ensemble projection regarding weekly COVID-19 deaths in the United States. The actual coverage of ensemble projections, based on weekly COVID-19 deaths across the projection period (April 26, 2024–April 28, 2025), is plotted against its expected coverage. Coverage measures the percentage of observations falling within a given prediction interval (e.g., for a 90% prediction interval, expected coverage is 90%). Coverage was calculated across all locations and projection weeks. The dashed diagonal lines represent the expectation for perfect accuracy (expected coverage is equal to actual coverage). When the solid black line is aboved the dashed line, the ensemble-based projections are overconfident (actual coverage is less than expected coverage). When the solid black line is below the dashed line, the ensemble-based projections are underconfident (actual coverage is more than expected coverage). To reflect the implemented CDC vaccine recommendation for 2024–2025, only projections for scenarios with vaccination recommendations to all individuals were included.

© 2025 Loo SL et al. *JAMA Network Open*.

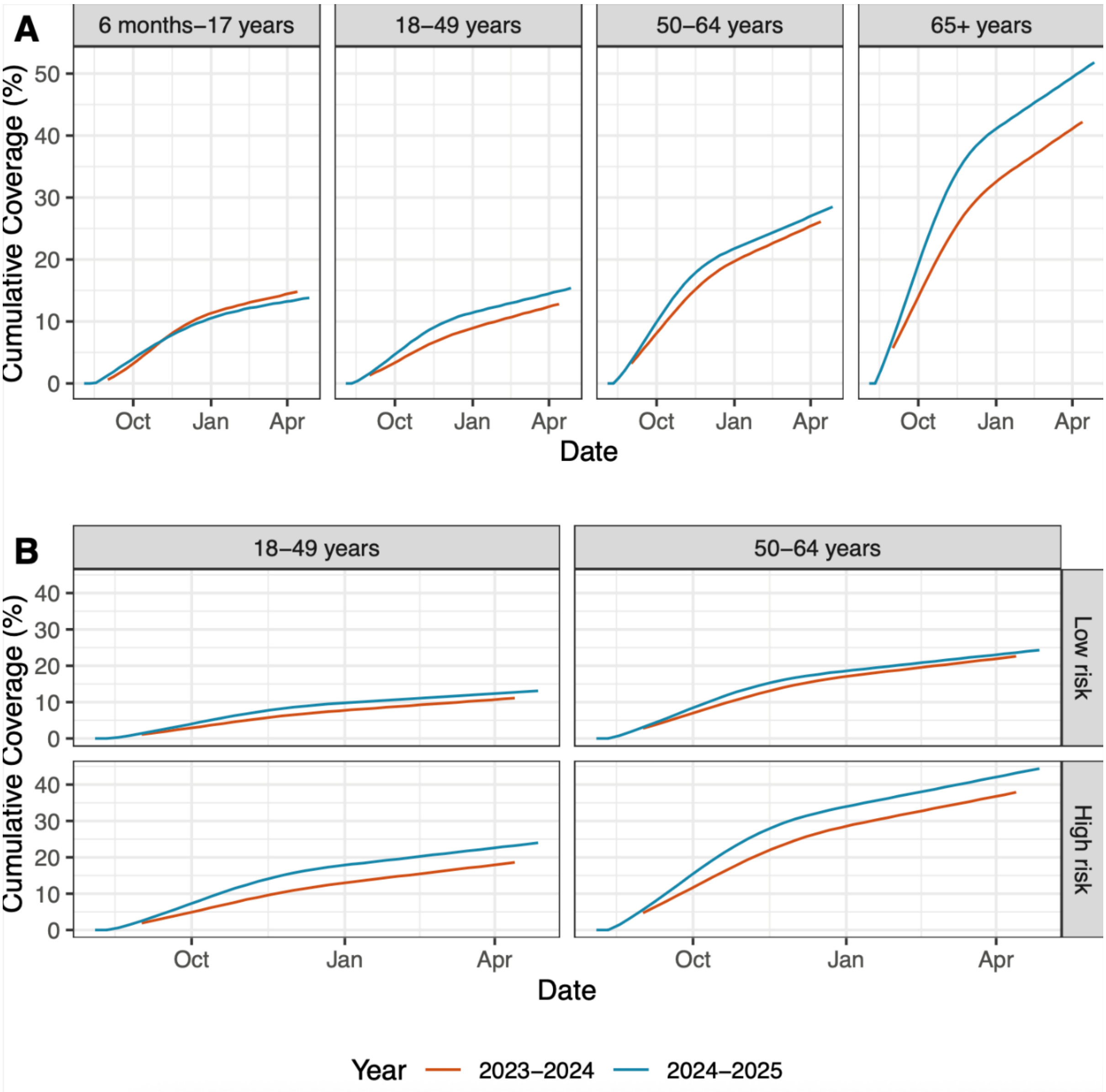

eFigure 4. Comparison between the assumed and observed uptake levels of COVID-19 vaccines at the national level in the United States. Red lines show the observed vaccine coverage in 2023–2024 by age and risk group[5], which was used as the assumed coverage for scenario projections. Blue lines represent the observed vaccine coverage during the projection period (2024–2025)[6].

© 2025 Loo SL et al. *JAMA Network Open*.

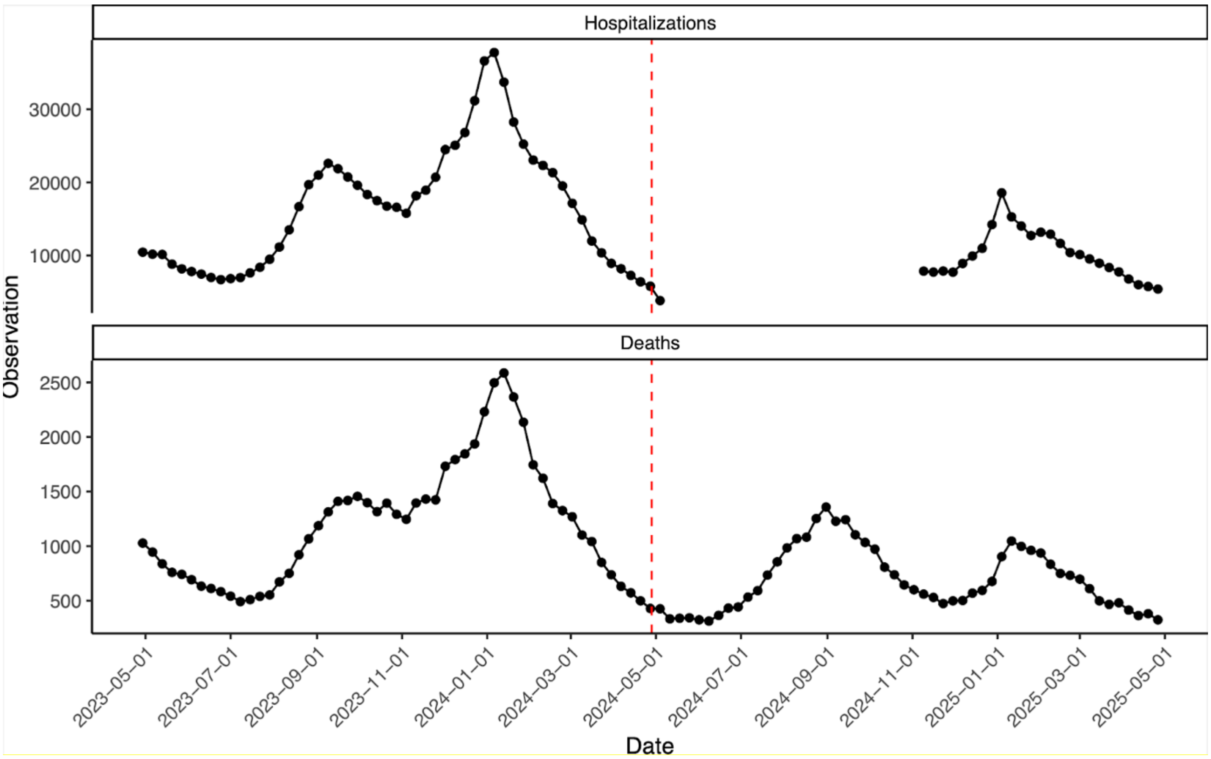

eFigure 5. Observed hospitalizations and death target data from April 28, 2023 to April 26, 2025 in the United States. Weekly COVID-19 hospitalizations and deaths at the national U.S. level[1,2], covering part of the calibration period (April 28, 2023–April 27, 2024) and the full projection period (April 28, 2024–April 27, 2025). The red dashed line indicates the start of the projection period. The gap in the hospitalization data corresponds to a period of underreporting, during which <75% of hospitals submitted data.

© 2025 Loo SL et al. *JAMA Network Open*.

eTable 1. Detailed description of individual models.

**Model name**			**JHU_UNC-flepiMoP**[7]	**MOBS-NEU-GLEAM_COVID**[8]	**NotreDame-FRED**[9]	**PSI-PROF**	**USC-SlkJalpha**[10]	**UT-ImmunoSERIS**[11]	**UVA-adaptive**[12]	**CFA-Scenarios**	**UNCC-hierbin**[13]
**Model type**			Age-specific compartmental model	Meta-population, age-specific, stochastic compartmental model	Agent-based model	Compartmental model	Age-specific compartmental model	Meta-population, compartmental model	Meta-population, compartmental model	Meta-population, compartmental model	Curve fitting and compartmental hybrid model
**Population structure**	Geographical resolution		State level	State level	Individual level	State level	State level	State level	State level	State level	State level
	Mobility data		Commuting data	Air-travel and commuting data	NA	NA	NA	NA	NA	NA	NA
	Mixing patterns among age groups		Homogeneous mixing	Contact matrices	Contact matrices	Contact matrices	Estimated	Contact matrices	Homogeneous mixing	Contact matrices	Homogeneous mixing
	Number of age groups (specific age-bands)		3 (0-17, 18-64, 65+)	10 (0-9, 10-19,20-24, 25-29, 30-39, 40-49, 50-59, 60-69, 70-79, 80+)	Individual ages	3 (0-17, 18-64, 65+)	3 (0-17, 18-64, 65+)	6 (0-4, 5-11, 12-17, 18-49, 50-64, 65+)	3 (0-17, 18-64, 65+)	4 (0-17, 18-49, 50-64, 65+)	3 (0-17, 18-64, 65+)
	Is importation from other countries considered?		No	No	No	No	No	No	No	No	No
**Natural history**	Is infectiousness age/risk factor-dependent?		No	No	No	No	No	No	No	No	No
	Is susceptibility age/risk factor-dependent?		No	No	Yes	No	Yes	Yes	No	No	No
	Infection Hospitalization Risk	From literature or estimated	Informed by literature, and estimated	Informed by literature	Informed by literature, and estimated	Estimated	Estimated	Informed by literature, and estimated	Informed by literature, and estimated	Estimated	Informed by literature, and estimated
		Age/risk factor-dependent?	Age- and risk factor-dependent	Age- and risk factor-dependent	Age- and risk factor-dependent	Age- and risk factor-dependent	Age-dependent	Age- and risk factor-dependent	Age- and risk factor-dependent	Age-dependent	No
**Antigenic drift of SARS-CoV-2**			Linear	Linear	Linear	Linear	Linear	Linear	Linear	Stepwise with frequent occurrence (a total of 4 introductions of new strains)	Linear

© 2025 Loo SL et al. *JAMA Network Open*.

Model name		JHU_UNC-flepiMoP	MOBS-NEU-GLEAM_COVID	NotreDame-FRED	PSI-PROF	USC-SlkJalpha	UT-ImmunoSERIS	UVA-adaptive	CFA-Scenarios	UNCC-hierbin
**Vaccine-derived immunity**	Age/risk factor-dependent?	No	No	No	No	Age-dependent	Age- and risk factor-dependent	No	No	No
	Is there protection against infection and/or transmissibility?	Infection only	Infection only	Infection only	Infection only	Infection only	Infection only	Infection only	Infection only	Infection only
	VE against infection on the date of release at population-level	50%	50%	40%	40%	90%	65%	65%	68%	65%
	Mechanism of protection	Leaky	Leaky	All-or-nothing	All-or-nothing	Leaky	Leaky	Leaky	Leaky	Leaky
	Does immune escape affect all protective effectiveness?	Yes	Yes	Yes	Yes	Yes	Yes	Yes	Yes	Yes
	Duration of immunity	Fitted, with a prior of 7 months half-time	Assumed 4 months half-time	Assumed 5 months (infection) and 12 months (hospitalization) half-times	Assumed 9 months half-time	Assumed, 4 months half-time to reach 20%	Assumed 3 months half-time	Assumed 4 months half-time	Fitted, 4 months half-time (only for infection).	Assumed 4 months half-time
**Infection-derived immunity**	Age/risk factor-dependent?	No	No	No	No	Age-dependent	Age- and risk factor-dependent	No	No	No
	Is there protection against infection and/or transmissibility?	Infection only	Infection only	Infection only	Infection only	Infection only	Infection only	Infection only	Infection only	Infection only
	Protection against hospitalization on the date of re-infection	Only impacts infection	Only impacts infection	Only impacts infection	Only impacts infection	Only impacts hospitalization	Impacts both infection and hospitalization	Impacts both infection and hospitalization	Only impacts hospitalization	Only impacts infection
	Mechanism of protection:	Leaky	Leaky	Leaky	Leaky	Leaky	Leaky	Leaky	Leaky	Leaky
	Does immune escape affect all protective effectiveness?	Yes	Yes	Yes	Yes	Yes	Yes	Yes	Yes	Yes
	Duration of immunity	Fitted, with a prior of 7 months half-time	Assumed 3 months half-time	Fitted, 7-13 months half-time	Fitted, 3-5 months half-time before moving to a partial immunity compartment	Assumed, 4 months half-time to reach 20%	Assumed 8 months half-time	Assumed 4 months half-time	Fitted, 4 months half-time (only for infection).	Assumed 4 months half-time
	Boosting effect from multiple infections?	No	No	No	No	Yes	No	No	No	No

© 2025 Loo SL et al. *JAMA Network Open*.

Model name		JHU_UNC-flepiMoP	MOBS-NEU-GLEAM_COVID	NotreDame-FRED	PSI-PROF	USC-SIkJalpha	UT-ImmunoSERIS	UVA-adaptive	CFA-Scenarios	UNCC-hierbin
**Seasonality Calibration process**	Is seasonality allowed to vary by geography?	Yes	Yes	Yes	Yes	Yes	Yes	Yes	Yes	Yes
	Are any exogenous (e.g., environmental factors or human movement) data considered in modeling seasonal forcing?	No	Air-travel data	Indoor activity level	No	No	Absoulte humidity	No	No	No
	Time period used for model calibration	03/19/2023-04/27/2024	10/29/2023-04/27/2024	04/23/2023-04/13/2024	05/06/2023-04/27/2024	Susceptibility: 01/25/2020-04/13/2024<br><br>Transmission: 01/01/2024-04/13/2024	09/25/2023-04/15/2024	10/28/2022-04/27/2024	02/11/2022-04/27/2024	04/14/2022-04/15/2024
	Is there an explicity observational model that consider under-reporting?	Yes	No	No	Yes	No	No	Yes	No	No
	Is any data outside target data used for calibration?	No	No	No	No	No	No	No	Seroprevalence and variant proportion surveillance data	No
	Fitting method	MCMC-like inference	Approximate bayesian computation	Maximum likehood	Latin hypercube	Least square	Least square	Golden section search & least square	MCMC	Recurrent neural network

© 2025 Loo SL et al. *JAMA Network Open*.

References

1. Weekly Hospital Respiratory Data (HRD) Metrics by Jurisdiction, National Healthcare Safety Network (NHSN) | Data | Centers for Disease Control and Prevention. February 11, 2025. Accessed February 11, 2025. https://data.cdc.gov/Public-Health-Surveillance/Weekly-Hospital-Respiratory-Data-HRD-Metrics-by-Ju/ua7e-t2fy/about_data

2. Provisional COVID-19 Death Counts by Week Ending Date and State | Data | Centers for Disease Control and Prevention. February 11, 2025. Accessed February 11, 2025. https://data.cdc.gov/NCHS/Provisional-COVID-19-Death-Counts-by-Week-Ending-D/r8kw-7aab/about_data

3. Hadfield J, Megill C, Bell SM, et al. Nextstrain: real-time tracking of pathogen evolution. *Bioinformatics*. 2018;34(23):4121-4123. doi:10.1093/bioinformatics/bty407

4. Chen C, Nadeau S, Yared M, et al. CoV-Spectrum: analysis of globally shared SARS-CoV-2 data to identify and characterize new variants. *Bioinformatics*. 2022;38(6):1735-1737. doi:10.1093/bioinformatics/btab856

5. COVID-19 Scenario Modeling Hub. COVID-19 Scenario Modeling Hub GitHub. July 28, 2023. https://github.com/midas-network/covid19-scenario-modeling-hub_archive

6. CDC. COVID-19 Vaccination Coverage and Intent for Vaccination, Adults 18 Years and Older, United States. May 7, 2025. https://www.cdc.gov/covidvaxview/weekly-dashboard/adult-vaccination-coverage.html

7. Lemaitre JC, Loo SL, Kaminsky J, et al. flepiMoP: The evolution of a flexible infectious disease modeling pipeline during the COVID-19 pandemic. *Epidemics*. 2024;47:100753. doi:10.1016/j.epidem.2024.100753

8. Davis JT, Chinazzi M, Perra N, et al. Cryptic transmission of SARS-CoV-2 and the first COVID-19 wave. *Nature*. 2021;600(7887):127-132. doi:10.1038/s41586-021-04130-w

9. Moore S, Cavany S, Perkins TA, España GFC. Projecting the future impact of emerging SARS-CoV-2 variants under uncertainty: Modeling the initial Omicron outbreak. *Epidemics*. 2024;47:100759. doi:10.1016/j.epidem.2024.100759

10. Ajitesh S. COVID-19 Forecasts for US states. https://scc-usc.github.io/ReCOVER-COVID-19/#/

11. Bouchnita A, Bi K, Fox SJ, Meyers LA. Projecting Omicron scenarios in the US while tracking population-level immunity. *Epidemics*. 2024;46:100746. doi:10.1016/j.epidem.2024.100746

12. Porebski P, Venkatramanan S, Adiga A, et al. Data-driven mechanistic framework with stratified immunity and effective transmissibility for COVID-19 scenario projections. *Epidemics*. 2024;47:100761. doi:10.1016/j.epidem.2024.100761

13. Chen S, Janies D, Paul R, Thill JC. Leveraging advances in data-driven deep learning methods for hybrid epidemic modeling. *Epidemics*. 2024;48:100782. doi:10.1016/j.epidem.2024.100782

© 2025 Loo SL et al. *JAMA Network Open*.
