# Supplementary material for: Scenario Projections of COVID-19 Burden in the US, 2024-2025
Source: JAMA Netw Open. 2025 Sep 18;8(9):e2532469. doi: 10.1001/jamanetworkopen.2025.32469 (PMC12447233; doi:10.1001/jamanetworkopen.2025.32469)
Supplement: Supplement 2. — Data Sharing Statement [file jamanetwopen-e2532469-s002.pdf]

Data Sharing Statement

Data

Data available: Yes
Data types: Data (not involving human participants), Other (please specify)
Additional Information: Model projections, target data and other details including vaccine coverage for scenarios are made publicly available in COVID-19 Scenario Modeling Hub Github repository (https://github.com/midas-network/covid19-scenario-modeling-hub)
How to access data: https://github.com/midas-network/covid19-scenario-modeling-hub
When available: beginning date: 06-28-2024

Supporting Documents

Document types: None

Additional Information

Who can access the data: Data is publicly available.
Types of analyses: For any purpose
Mechanisms of data availability: Data is publicly available to all
